# Age and sex subgroups vulnerable to copycat suicide: evaluation of nationwide data in South Korea

**DOI:** 10.1038/s41598-019-53833-8

**Published:** 2019-11-21

**Authors:** Hahn Yi, Jeongeun Hwang, Hyun-Jin Bae, Namkug Kim

**Affiliations:** 1 Asan Institute for Life Sciences, Asan Medical Center, Seoul, Republic of Korea; 2 Department of Medicine, University of Ulsan College of Medicine, Seoul, Republic of Korea; 3 Department of Convergence Medicine, University of Ulsan College of Medicine, Asan Medical Center, Seoul, Republic of Korea; 4 Department of Pulmonary and Critical Care Medicine, University of Ulsan College of Medicine, Asan Medical Center, Seoul, Republic of Korea

**Keywords:** Psychiatric disorders, Epidemiology, Risk factors

## Abstract

Media reports of a celebrity’s suicide may be followed by copycat suicides, and the impact may vary in different age and sex subgroups. We proposed a quantitative framework to assess the vulnerability of age and sex subgroups to copycat suicide and used this method to investigate copycat suicides in relation to the suicides of 10 celebrities in South Korea from 1993 to 2013. By applying a detrending model to control for annual and seasonal fluctuations, we estimated the expected number of suicides within a copycat suicide period. The copycat effect was assessed in two ways: the magnitude of copycat suicide by dividing the observed by the expected number of suicides, and the mortality rate by subtracting the expected from the observed number of suicides. Females aged 20–29 years were the most vulnerable subgroup according to both the magnitude of the copycat effect (2.31-fold increase over baseline) and the mortality rate from copycat suicide (22.7-increase). Males aged 50–59 years were the second most vulnerable subgroup according to the copycat suicide mortality rate (20.5- increase). We hope that the proposed quantitative framework will be used to identify vulnerable subgroups to copycat effect, thereby helping devise strategies for prevention.

## Introduction

Suicide is one of the most significant public health problems worldwide; the annual global age-standardised suicide rate was 10.5 per 100,000 in 2016^1^. The situation is more severe in South Korea, which has the second highest suicide rate of all the Organization for Economic Cooperation and Development countries; a suicide rate of 25.8 per 100,000 was reported in 2015^2^, and suicide was the fifth most common cause of death in South Korea in 2018. Suicide was the leading cause of death in those aged 10 to 39 years and the second leading cause of death in those aged 40 to 59 years^3^. Suicide is a complex phenomenon that is influenced by personal characteristics^4,5^ and various socioenvironmental factors^6,7^.

Copycat suicide, often called the Werther effect, is an imitative suicidal behaviour that occurs after exposure to another suicide. Especially, exposure to media reports of a celebrity’s suicide exert considerable copycat effect on at-risk individuals^8–10^. Documented typical copycat suicide patterns are composed of both mass and point clusters^11,12^. The mass clusters mainly exhibit a one-to-many transfer mode, while the point clusters are propagated to nearby individuals. More specifically, the mass clusters can be used to define the upsurge in suicide frequency that is particularly caused by mass media, which can trigger copycat suicides^13–15^. Copycat suicide is expected to become more common in our increasingly connected world. Increases in internet news media, personal broadcasting, online communities and social network services in recent years have provided a highly connected matrix whereby provocative news articles, including reports of celebrity suicide, can travel rapidly and widely^16^. Globalisation of fandom may also expose a larger population to the risk of copycat suicide.

Some studies have reported the mass clusters effect of copycat suicide following a celebrity suicide^17–20^. However, few studies have focused on the associations between celebrity suicides and copycat suicide subgroups. Myung *et al*. studied the sex and age subgroups susceptible to copycat suicide by comparing the number of suicides 1 month pre-event with those 1 month post-event^21^. Using data from a national population-based database in South Korea, the authors found that younger females are more likely to commit suicide following a celebrity suicide than other age and sex subgroups, and Jang *et al*. reported a similar result^19^. Park *et al*. performed a subgroup analysis of copycat suicide in South Korea and found that celebrity suicides had significant impacts on suicide rates among persons of the same sex or the same age as the celebrity^22^. None of these studies provided a quantitative framework to assess the magnitude of copycat suicide according to age and sex subgroups.

Effective prevention of copycat suicide requires more specific identification of vulnerable subgroups. A quantitative framework to assess the magnitude of copycat suicide and the mortality rate from copycat suicide is urgently needed to determine the relationship between copycat suicide in different age and sex subgroups and celebrity suicide cases.

This study aimed to provide a quantitative framework to assess the copycat suicide effect and to identify age and sex subgroups vulnerable to copycat suicide in South Korea using this framework. We applied a linear regression detrending model to control for confounding effects and to estimate the expected number of suicides. We proposed a measure of the magnitude of copycat suicide calculated by dividing the observed by the expected number of suicides. In addition, we proposed a measure of the mortality rate from copycat suicide calculated by subtracting the expected from the observed number of suicides. By comparing these two measures between different age and sex subgroups, we identified the subgroups that were more vulnerable to commit copycat suicide in South Korea.

## Results

In accordance with the inclusion and exclusion criteria described in the Materials and Methods, 10 celebrity suicide cases and six celebrity death cases with nonsuicidal causes were analysed. The celebrity death cases with nonsuicidal causes were included as a control group. Table 1 shows the name, age at death, sex, event date, time window for copycat suicides determined per celebrity suicides on the basis of the pre-defined exponential model^17^, career and cause of death for the 10 suicidal and six nonsuicidal celebrity death cases. For all cases, there were a small number of observations for the age subgroups ≥70 years and <10 years, so only the age subgroups between 10 and 69 years were compared.

**Table 1:** Characteristics of celebrity death events.

Case	Name	Age at death (yr)	Sex	Event date (mm/dd-yyyy)	Time window of interest for copycat suicides	Career	Cause of death
Celebrity 1	J. Choi	39	Female	10/02-2008	10/03-2008–10/22-2008	Actor	Suicide
Celebrity 2	J. Jang	29	Female	03/07-2009	03/07-2009–03/26-2009	Actor	Suicide
Celebrity 3	E. Lee	24	Female	02/22-2005	03/02-2005–03/12-2005	Actor	Suicide
Celebrity4	D. Jeong	26	Female	02/10-2007	02/10-2007–02/18-2007	Actor	Suicide
Celebrity 5	J. Song	29	Female	05/23-2011	05/23-2011–05/28-2011	Anchor	Suicide
Celebrity 6	M. Roh	62	Male	05/23-2009	05/26-2009–06/05-2009	Politician	Suicide
Celebrity 7	M. Chung	54	Male	08/04-2005	08/04-2003–08/23-2008	Businessman	Suicide
Celebrity 8	Y. Park	32	Male	06/30-2010	07/01-2010–07/16-2010	Actor	Suicide
Celebrity 9	J. Choi	39	Male	03/29-2010	04/04-2010–04/10-2010	Actor	Suicide
Celebrity 10	J. Ahn	36	Male	09/08-2008	09/15-2008–09/20-2008	Actor	Suicide
Celebrity 11	A. Kim	74	Male	08/12-2010	08/15-2010–08/20-2010	Fashion	Disease
Celebrity 12	D. Choi	53	Male	09/14-2011	09/15-2011–09/21-2011	Sports	Disease
Celebrity 13	H. Kim	26	Female	01/10-2007	01/10-2007–01/13-2007	Entertainment	Accident
Celebrity 14	E. Lee	27	Male	08/21-2008	08/24-2008–08/27-2008	Actor	Accident
Celebrity 15	S. Lim	29	Male	04/02-2008	04/03-2008–04/08-2008	Entertainment	Disease
Celebrity 16	Y. Lim	24	Male	02/11-2013	02/11-2013–02/19-2013	Entertainment	Disease

**Figure 1:**
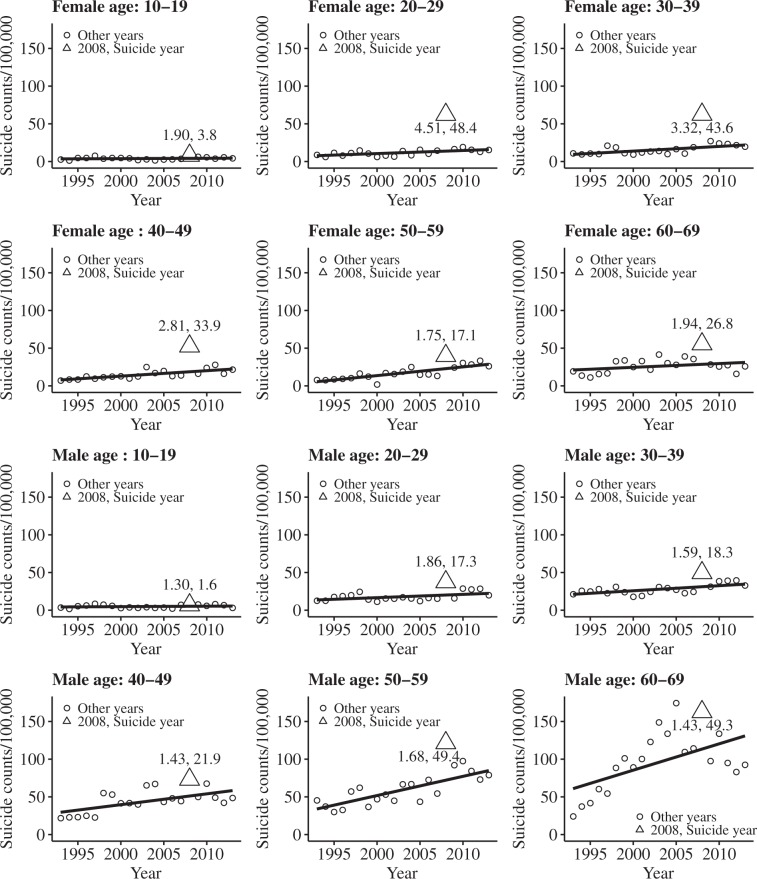
Magnitude of copycat suicides in female (upper two rows) and male (bottom two rows) age subgroups following Celebrity 1’s suicide. The expected number of suicides was estimated by detrending linear regression models (solid lines). The observed numbers of suicides during the copycat period of all years except 2008 are shown by circles. The observed numbers of suicides during the copycat suicide period of 2008 are shown by triangles. The ratios of the observed to the expected numbers of suicides are shown as numbers above (or below) the triangles.

For each celebrity suicide case, age subgroup and sex subgroup, the magnitude of copycat suicide and the mortality rate from copycat suicide were calculated. For instance, Celebrity 1, who was a popular female actor, committed suicide on 2 October 2008, inducing copycat suicides during the interval from October 3 to 22, 2008 (Table 1). The numbers of all suicides occurring during this period were counted for each year between 1993 and 2013, except for 2008, and a detrending model was constructed for each age and sex subgroup to deduce the expected number of suicides. Figure 1 shows the magnitudes and mortality rates of copycat suicides in each subgroup for the Celebrity 1 case, calculated by dividing the observed by the expected number of suicides and subtracting the expected from the observed number of suicides, respectively. For example, between October 3 and 22, 2008, females aged 10–19 years committed 1.905-fold more suicides than expected, and the mortality rate from suicide in this subgroup was increased by 3.801.

**Figure 2:**
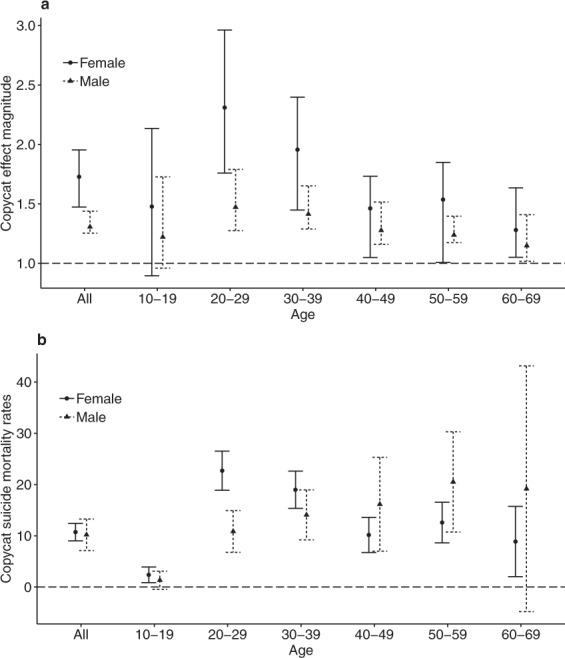
The magnitude of copycat suicide and the mortality rate from copycat suicide according to age and sex subgroups. (**a**) Copycat effect magnitude and (**b**) copycat suicide mortality rate and 95% confidence intervals according to age (total, 10–19, 20–29, 30–39, 40–49, 50–59, 60–69 years) and sex (females, circles and solid lines; males, triangles and dashed lines) for the total of 10 celebrity suicide cases (C1–C10).

For each celebrity suicide case and each age and sex subgroup, the magnitude of copycat suicide (Table 2, Fig. 2a) and the mortality rate from copycat suicide (Table 3, Fig. 2b) were measured and represented by 95% confidence intervals (CIs). No increases in suicides occurred after the deaths of the control non-suicidal celebrities (results not shown).

**Table 2:** Copycat effect magnitude for Celebrities 1–10: 95% confidence intervals for the ratio of observed to expected number of suicides.

Age (yr)	Sex	C1	C2	C3	C4	C5	C6	C7	C8	C9	C10	Total
10–19	Male	1.04–1.74	1.33–1.91	0.70–0.99	0.82–1.51	1.02–2.89	1.73–3.02	0.69–1.00	0.76–1.49	0.37–0.80	1.02–2.69	0.96–1.73
	Female	1.54–2.49	1.39–2.05	1.55–2.53	1.47–2.81	0.48–0.93	0.97–1.80	1.12–1.69	2.02–4.05	0.94–4.00	0.38–0.85	0.90–2.13
20–29	Male	1.62–2.17	1.36–1.86	1.20–1.49	1.39–1.83	1.02–2.89	1.08–1.55	1.32–1.76	1.34–1.97	1.30–2.05	0.70–1.03	1.28–1.79
	Female	3.97–5.21	1.37–2.31	2.04–3.47	3.32–5.10	1.26–2.19	2.21–3.00	1.34–1.79	1.81–2.68	1.26–2.44	1.31–1.92	1.76–2.96
30–39	Male	1.45–1.76	0.98–1.29	1.46–1.88	1.18–1.60	1.50–2.04	0.95–1.31	1.13–1.40	1.48–1.84	1.40–2.03	0.86–1.28	1.29–1.65
	Female	2.91–3.87	2.22–3.03	1.50–1.99	3.42–4.57	0.79–1.53	1.43–2.05	1.13–1.51	1.77–2.55	1.05–1.50	1.34–1.95	1.49–2.40
40–49	Male	1.25–1.67	0.86–1.10	1.55–1.98	1.00–1.44	0.83–1.23	1.14–1.53	1.68–2.14	0.95–1.42	1.21–1.86	0.79–1.15	1.16–1.52
	Female	2.49–3.23	1.14–1.49	1.21–1.61	1.75–2.49	0.72–1.00	0.96–1.30	1.57–1.96	1.36–1.90	1.24–1.75	0.89–1.30	1.05–1.73
50–59	Male	1.53–1.88	0.95–1.13	1.24–1.47	1.01–1.36	1.07–1.54	1.26–1.53	1.62–1.90	1.10–1.33	1.04–1.37	0.70–0.92	1.17–1.40
	Female	1.55–2.01	1.51–1.94	1.29–1.57	1.63–2.43	1.64–2.65	1.14–1.60	1.70–2.22	0.99–1.49	1.18–1.59	0.80–1.28	1.00–1.85
60–69	Male	1.22–1.74	0.79–1.12	1.23–1.66	0.66–0.90	0.61–1.05	1.04–1.54	1.57–2.02	0.88–1.35	0.95–1.65	1.29–2.02	1.00–1.41
	Female	1.65–2.36	0.85–1.18	1.14–1.71	1.33–1.94	0.45–0.79	1.00–1.52	1.60–2.15	1.23–1.95	0.88–1.50	0.93–1.62	1.05–1.64
Age	Male	1.44–1.55	1.02–1.21	1.34–1.55	1.08–1.30	1.13–1.41	1.21–1.44	1.45–1.69	1.20–1.45	1.27–1.54	0.93–1.19	1.25–1.44
total	Female	2.70–3.17	1.51–1.93	1.61–1.98	2.60–3.10	0.99–1.36	1.42–1.77	1.44–1.69	1.55–2.01	1.24–1.58	1.12–1.43	1.47–1.95
Total		1.65–1.88	1.19–1.40	1.44–1.65	1.54–1.81	1.09–1.38	1.45–1.70	1.28–1.53	1.45–1.70	1.32–1.59	1.27–1.53	1.42–1.57

**Table 3:** Copycat effect mortality rates for Celebrities 1–10: 95% confidence intervals for the observed minus the expected number of suicides.

Age (yr)	Sex	C1	C2	C3	C4	C5	C6	C7	C8	C9	C10	Total
10–19	Male	0.3–2.9	2.4–4.7	−2.8–0.0	−1.2–1.8	0.2–5.4	6.4–10.2	−2.0–0.0	−1.6–1.7	−6.2–−1.0	0.2–5.5	−0.4–3.1
	Female	2.8–4.8	2.5–4.6	3.9–6.6	2.3–4.6	−5.2–−0.3	−0.2–3.2	0.6–2.5	6.1–9.1	−0.4–4.5	−3.9–−0.4	0.8–3.9
20–29	Male	14.4–20.2	8.4–14.6	4.8–9.6	9.2–14.9	12.1–26.3	2.2–10.9	7.0–12.5	9.3–18.2	9.5–21.3	−9.1–0.6	6.8–14.9
	Female	46.5–50.2	8.1–17.1	21.1–29.4	44.0–50.6	7.2–19.0	28.1–34.2	5.0–8.7	14.7–20.6	7.5–21.7	6.6–13.4	18.9–26.5
30–39	Male	15.3–21.3	−0.8–8.0	17.5–26.0	6.5–16.2	22.5–34.3	−2.0–10.0	4.2–10.4	17.7–25.0	17.0–30.2	−6.0–7.9	9.2–19.0
	Female	40.9–46.2	25.1–30.6	10.2–15.2	45.6–50.4	−7.8–10.0	11.1–18.9	2.2–6.5	16.6–23.1	1.4–10.2	7.8–15.0	15.3–22.6
40–49	Male	14.6–29.2	−8.3–4.6	31.5–44.1	0.1–17.9	−14.8–13.8	10.3–27.7	34.3–45.1	−3.3–19.6	15.3–40.4	−13.8–6.5	7.0–25.3
	Female	31.4–36.3	3.4–9.0	4.6–10.0	14.9–20.7	−10.5–0.0	−1.1–6.6	10.7–14.6	9.3–16.7	7.6.–16.6	−3.0–5.4	6.7–13.6
50–59	Male	41.9–56.8	−4.5–9.3	19.8–32.4	0.5–21.4	8.0–43.5	25.9–44.0	42.4–52.6	9.4–26.0	5.0–33.7	−30.6–−6.2	10.7–30.3
	Female	14.1–20.2	13.0–18.6	6.0–9.6	13.5–20.5	22.0–35.0	4.7–14.1	13.0–17.4	−0.3–9.4	6.3–15.3	−6.1–5.5	8.6–16.6
60–69	Male	29.4–69.2	−27.8–11.6	25.2–54.0	−39,4–−9.8	−76.4–6.4	6.6–58.8	58.5–81.3	−18.3–34.7	−9.6–69.7	43.1–96.9	−4.84–43.1
	Female	21.7–31.9	−5.1–4.5	4.3–15.0	11.1–21.4	−28.2–−6.1	0.1–13.2	16.1–23.0	9.2–23.8	−6.0–15.0	−2.9–15.6	2.0–15.7
Age	Male	14.2–18.4	0.7–6.1	11.0–15.3	2.4–7.8	6.2–15.3	8.3–15.0	12.2–16.0	7.4–13.8	11.6–19.1	−2.8–5.7	7.1–13.3
total	Female	23.8–25.9	8.2–11.6	8.6–11.2	21.0–23.1	−0.3–6.0	7.7–11.5	5.1–7.2	8.9–12.6	5.0–9.3	2.1–5.7	9.0–12.4
Total		19.3–22.9	4.7–8.6	10.1–13.0	11.8–15.2	3.2–10.4	8.7–11.5	8.2–13.1	8.7–11.5	8.3–13.0	8.6–13.9	8.3–12.7

### The magnitude of copycat suicide

The magnitude of copycat suicide for the total of 10 celebrity cases was highest in females aged 20–29 years (95% CI, 1.76–2.96), followed by females aged 30–39 years (1.45–2.40) (Table 2, Fig. 2a). The magnitude of copycat suicide was generally higher for females than for males in the same age subgroup.

### The mortality rate from copycat suicide

The mortality rate from copycat suicide for the total of 10 celebrity cases was also highest in females aged 20–29 years (95% CI, 8.9–26.5) (Table 3, Fig. 2b). Males aged 40–49 years (95% CI, 7.0–25.9) and 50–59 years (95% CI, 10.7–30.3) had copycat suicide mortality rates that were comparable to, or slightly less than, those of females aged 20–29 and 30–39 years (Table 3). There was no general tendency for female subgroups to be more vulnerable than male subgroups according to the mortality rate from copycat suicide.

### Sex of the celebrity

**Figure 3:**
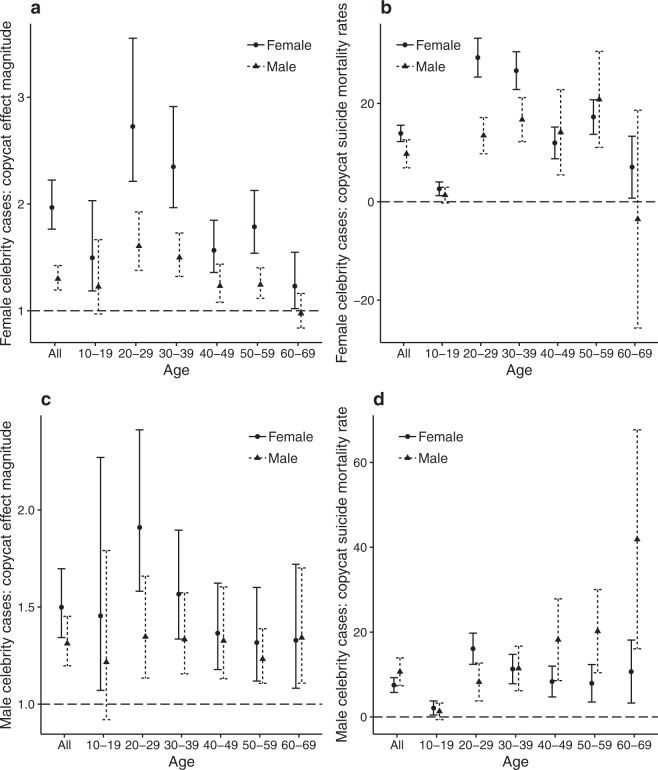
Relationship between the sex of the suicided celebrity and the sex of the person who committed copycat suicide. (**a**) Copycat effect magnitude and (**b**) copycat suicide mortality rate and 95% confidence intervals according to age (total, 10–19, 20–29, 30–39, 40–49, 50–59, 60–69 years) and sex (females, circles and solid lines; males, triangles and dashed lines) for female (C1–C5) and male (C6–C10) celebrity suicide cases (**c,d**).

To investigate whether there was a relationship between the sex of the celebrity and the sex of the person who committed copycat suicide, the data for female (C1–C5) and male (C6–C10) celebrities were analysed separately for their effect on the magnitude and mortality rate of copycat suicides. Among females who committed copycat suicide, the effect was greater when the celebrity was also female (Fig. 3a,b). Among males who committed copycat suicide, there was no clear relationship with the sex of the celebrity (Fig. 3c,d).

### Age of the celebrity

**Figure 4:**
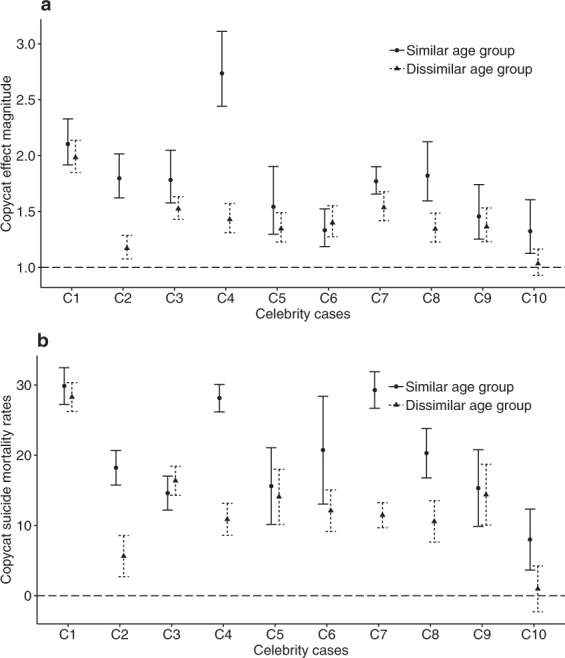
Relationships between the age of the celebrities and the age of person who committed copycat suicide. (**a**) Copycat effect magnitude and (**b**) copycat suicide mortality rate and 95% confidence intervals for victims of similar age group (from 5 years younger to 5 years older than the celebrity; circles and solid lines) and dissimilar age group (triangles and dashed lines) for each of the celebrity cases (C1–C10).

To investigate whether there was a relationship between the age of the celebrity and the age of the person who committed copycat suicide, those who committed copycat suicide were classified according to whether they belonged to a similar age group to the celebrity (from 5 years younger to 5 years older than the celebrity) or a dissimilar age group. In most cases, those who belonged to a similar age group to the celebrity were more vulnerable to copycat suicide (Fig. 4).

## Discussion

We have proposed a new method to quantify the vulnerability of different age and sex subgroups to copycat suicide and used this method to investigate the effect of 10 cases of celebrity suicide in South Korea from 1993 to 2013. Application of a linear regression detrending distinguished the copycat suicide effect from variability resulting from annual trends and seasonal fluctuations. The magnitude and the mortality rate are the two proposed indices of the effect of copycat suicide, and they are complementary to each other. The magnitude of copycat suicide for each celebrity case and each subgroup was assessed by dividing the observed by the expected number of suicides. The magnitude indicates the extent to which a celebrity suicide provoked the members of a subgroup to commit suicide compared with baseline. The mortality rate from copycat suicide, estimated by subtracting the expected from the observed number of suicides, indicates the increase in the number of suicides due to the copycat effect.

The two notably vulnerable subgroups were females aged 20–29 years and males aged 50–59 years. Females aged 20–29 years were the most vulnerable subgroup in terms of both the magnitude of the copycat effect (2.31-fold increase over baseline; 95% CI, 1.76–2.96) (Table 2 and Fig. 2a) and the mortality rate from copycat suicide (22.7-fold increase; 95% CI, 18.9–26.5) (Table 3 and Fig. 2b) for the total of 10 celebrity suicide cases. Males aged 50–59 years were the second most vulnerable subgroup in terms of the mortality rate from copycat suicide (20.5-fold increase; 95% CI, 10.7–30.3) (Table 3 and Fig. 2b), but not in terms of the magnitude of the copycat effect (1.29-fold increase; 95% CI, 1.17–1.40) (Table 2 and Fig. 2a). This difference may have resulted from the higher baseline suicide mortality rate among males aged 50–59 years than among females aged 20–29 years. For example, the expected baseline suicide mortality rate for females aged 20–29 years was 13.8, whereas that for males aged 50–59 years was 72.1 (Fig. 1).

Female subgroups generally exhibited greater magnitudes of copycat suicide than did male subgroups of the same age, although several exceptions were observed. In female celebrity suicide cases C1, C2, C3, C4 and C5, the magnitude of copycat suicide was larger in female subgroups than in male subgroups for all ages (Table 2). These results are largely in agreement with previous findings that females are more likely to commit copycat suicide than males^21–24^ and that stories of female celebrities’ suicide exert a greater influence on female copycat suicide victims than those of male celebrities^21^.

Previous studies that compared the copycat suicide effect between same-sex and opposite-sex celebrities found that the same-sex subgroup was more vulnerable than the opposite-sex subgroup^19,22,25,26^. However, one study found that the same-sex effect on copycat suicide was unclear^27^. In our study, female celebrity suicides exerted greater copycat effects on females than on males in terms of both the magnitude of the copycat effect (95% CI, 1.76–2.22 for females and 1.19–1.42 for males) and the copycat suicide mortality rate (95% CI, 12.3–15.6 for females and 6.9–12.6 for males) (Fig. 3a,b). The effect was most prominent in females aged 20–29 years and 30–39 years, considering both magnitude and mortality rate. Males did not exhibit as clear a pattern as females (Fig. 3c,d)^19,22,25–27^. The greater vulnerability of females to copycat suicide may be explained by sex differences in psychopathological vulnerability. Some mood disorders that may lead to suicide, such as depression^28,29^, eating disorders^30^ and anxiety disorder^31^, are more prevalent in females than in males. A tendency for females to be more empathetic to other people’s stories^32,33^ may have played a role in augmenting suicidal ideation when they were exposed to media reports of a celebrity’s suicide. Stack^23,34^ suggested that females may be more reliant than males on an external party to legitimise suicide and that exposure to media reports of suicide could have served the legitimacy function. The specific mechanisms that determine why females are more prone to same-sex copycat suicide than males deserve further investigation.

The conjecture that copycat suicide is more likely when the celebrity and the person who commits copycat suicide are of similar age has been explored in previous studies^19,22,25,26,34–37^. Stack proposed in a differential identification model of copycat suicide that imitative suicidal behaviour is more prevalent in people sharing similar social characteristics and showed that being of similar age with the person committing suicide was a risk factor for copycat suicide^38^. Our study largely supports the similar age conjecture (Fig. 4), in accordance with previous studies^19,22,25,26,34–36^, but with a couple of exceptions. In magnitude of copycat effect (Fig. 4a), one exception was Celebrity 6 who was a 62 years old politician. We presume that this exception might be due to a gradual build-up of his negative reputation in middle-aged males in South Korea since his presidency. In Fig. 4b, regarding the mortality rate from copycat suicide, one exception was Celebrities 3, where we found similar or lower copycat suicide mortality rate in the similar age group than in the dissimilar age group. Celebrity 3 was a 24 years old female, but we found males aged 30–39 and males aged 40–49 exhibited larger copycat suicide mortality rate than males aged 20–29 (data not shown).

In the cases of three male actors, Celebrities 8, 9 and 10, high popularity seemed to exert a strong copycat suicide effect. Celebrity 8 frequently played major characters in television dramas and films until the time of his suicide, and the public had had many opportunities to feel empathy for his on-screen characters. On the other hand, although the other two male celebrities had been popular in the past, they were rarely exposed to the public at the time they committed suicide, which could explain the smaller number of copycat suicides associated with them compared with those associated with recently active celebrities. The suicide of a celebrity who has been frequently exposed to the public just before committing suicide is known to increase the magnitude of copycat suicide^18^.

From the hypothesis suggested by Stack in his review article in 2005^23^, we inferred that a businessman’s suicide would exert less copycat suicide effect than the suicide of an entertainer or politician. In our study, however, Celebrity 7, a businessman, did not exert an inferior copycat effect to that of other celebrities, in terms of both magnitude and mortality rate. Celebrity 7 was the heir to the founder of one of the most notable enterprises and had received extensive media attention. The fact that more news articles about Celebrity 7’s case were published might explain our finding. It has been suggested that increased media exposure is associated with more copycat suicides^17^.

We found no significant copycat suicide effects following the deaths of celebrities from disease or accidents (Table 1, Celebrities 11–16). The loss of a celebrity saddens the public, so we included Celebrities 11–16 as positive controls, speculating that the sense of loss may have led to suicides. Despite comparable media coverage in the month after their deaths, we found no significant increase in suicide counts in response to the deaths of Celebrities 11–16, which implies that the sense of loss without suicide commitment exerted negligible effects on copycat suicide behaviour. Queinec *et al*. conducted a positive control analysis in France that included seven celebrities who died from accidents or murder and found only limited copycat suicide effects^18^.

In spite of the strength of our study, which is based on nationwide, extensive, all-death census data for up to 21 years, there are limitations to be mentioned. Our analysis is geographically and culturally confined to South Korea, limiting generalizability to other parts of the world. In South Korea, a guideline for reporting suicides in media was first published in 2004 and updated to version 2.0 in 2013 and subsequently to version 3.0 in 2018^6^. The guideline does not grossly differ from the WHO guideline^39^. In reality, however, researches^6,40,41^ found out that the media reports did not comply with the guideline sufficiently regardless of their study years. Reporting attitude may have varied among the celebrity suicide cases, but currently we do not have evidence for such variations.

Another major limitation is the lack of information on individual suicide victims: his or her extent of media exposure and imitative motivation in committing suicide. These are essential pieces of information to reveal the underlying mechanism of vulnerability to copycat suicide, which is beyond the reach of the current study. Nevertheless, these limitations do not preclude us from suggesting a quantitative framework to assess and compare copycat suicide vulnerability between multiple age- and sex- subgroups and from identifying the most vulnerable subgroups.

## Conclusion

This study provides a quantitative framework for assessing the copycat suicide effect. We demonstrated a detrending method to reveal the copycat effect while controlling for annual and seasonal fluctuations in suicide counts. Applying this suggested framework to the South Korean national mortality statistics enabled us to find out that all age and sex subgroups suffered from copycat effect, and females aged 20–29 years subgroup was the most vulnerable to copycat effects. We hope that the proposed framework will facilitate further suicide prevention studies and strategies to ameliorate the copycat suicide effect.

## Materials and Methods

### Ethical approval

Ethical approval was not required, because this study was performed using a publicly accessible national epidemiology database.

### Suicide mortality statistics

Mortality microdata published by the Korean National Statistics Office were used to extract suicide mortality statistics from 1993 to 2013. In each of the microdata, causes of deaths are classified by the International Classification of Disease (ICD)-10 code. Cases for suicide mortality were extracted by identifying X60–X84 codes in the cause-of-death field in each microdata. The extracted suicide mortality data were again classified according to the celebrity’s date of death, sex and age at death. Mid-year population data provided by the Korean Statistical Information Service (KOSIS, http://kosis.kr) were used to compute the number of suicide deaths per 100,000.

### Celebrity suicide events

Within the 1993 to 2013 study period, 10 celebrity suicide events were included in the present study. The selection process was largely the same as that in a previous study^17^ that identified 15 celebrity suicides by the number of articles that were published by the top three newspapers in Korea (Chosun Ilbo, Chungang Ilbo and Donga Ilbo) within the first 7 days after the suicide. Among the most published 15 celebrities, the top five female and male celebrities were included according to the numbers of published news articles (Table 1, female Celebrities 1–5 and male Celebrities 6–10).

As positive controls, six other celebrity deaths caused by disease or accidents were selected (Table 1, Celebrities 11–16). With the use of the Naver news search engine, which has the largest market share in South Korea, celebrity events in the control group were selected according to the number of news reports within 1 month after the celebrity’s death.

### Assessing copycat suicide effects

The methods outlined in our previous study^17^ were followed to define and estimate the time window for copycat suicides. In brief, the time window of the copycat suicide effect was determined per celebrity suicides on the basis of the pre-defined exponential model fitting^17^. To estimate the magnitude of copycat following a celebrity suicide event, it was first assumed that there are baseline suicide mortality levels. This baseline suicide level can vary due to seasonal or year-to-year fluctuations or to unknown causes other than copycat suicide. Therefore, the baseline suicide mortality rate within a specific period was estimated and detrended using a linear regression model, which was based on the number of suicide events during the period for every year from 1993 to 2013, except the year to which the period of interest belonged. A low correlation between unemployment and suicide rates during the study period provided a reason to ignore the unemployment rate in this study.

Copycat suicide effects were quantified by two measures, the copycat effect magnitude and the copycat suicide mortality rate. The magnitude of the copycat effect of a celebrity suicide event was calculated by dividing the observed by the expected number of suicides from the detrending method. The copycat suicide mortality rate was calculated by subtracting the expected from the observed number of suicides. Both the copycat effect magnitude and the copycat suicide mortality rate were calculated for age (10–19, 20–29, 30–39, 40–49, 50–59 and 60–69 years) and sex (male/female) subgroups. Statistical analyses were performed using R statistical software version 3.5.1 (R Foundation for Statistical Computing, Vienna, Austria) and Python version 3.7.1.

## Data Availability

The data used in this study are available from the following public databases: Mortality microdata, Korean National Statistics Office, http://mdis.kostat.go.kr; and Mid-year population, KOSIS, http://kosis.kr.
